# A metagenomic study of the microbial communities in four parallel biogas reactors

**DOI:** 10.1186/s13068-014-0146-2

**Published:** 2014-10-14

**Authors:** Linn Solli, Othilde Elise Håvelsrud, Svein Jarle Horn, Anne Gunn Rike

**Affiliations:** Bioforsk, Norwegian Institute for Agricultural and Environmental Research, Frederik A. Dahls vei 20, 1432 Ås ᅟ, Norway; Department of Microbiology, Oslo University Hospital, P.O. Box 4950, Nydalen, 0424 Oslo, Norway; Department of Chemistry, Biotechnology and Food Science, Norwegian University of Life Sciences, P.O. Box 5003, N-1432, Ås ᅟ, Norway; Norwegian Geotechnical Institute, Sognsveien 72, P.O. Box 3930, Ullevål Stadion, N-0806 Oslo, Norway

**Keywords:** Anaerobic digestion, Syntrophic oxidation, Metagenomic, Biogas, Taxonomic structure, Biofuel, Biorefinery, Methane

## Abstract

**Background:**

Biogas is a renewable energy carrier which is used for heat and power production or, in the form of purified methane, as a vehicle fuel. The formation of methane from organic materials is carried out by a mixed microbial community under anaerobic conditions. However, details about the microbes involved and their function are limited. In this study we compare the metagenomes of four parallel biogas reactors digesting a protein-rich substrate, relate microbiology to biogas performance, and observe differences in these reactors’ microbial communities compared to the original inoculum culture.

**Results:**

The biogas process performance during the startup phase of four parallel continuous stirred tank reactors (designated R1, R2, R3, and R4) co-digesting fish waste and cow manure was studied. The microbial composition of the inoculum (day 0) and the four reactors at day 59 was studied and compared using 454 FLX Titanium pyrosequencing. In the inoculum and the reactor samples, the Bacteria *Clostridium* and *Syntrophomonas* were highly abundant, and the dominating methanogen was the hydrogenotrophic *Methanoculleus*. Syntrophic prokaryotes frequently found in biogas reactors with high concentrations of ammonium and volatile fatty acids were detected in all samples. The species *Candidatus Cloacimonas acidaminovorans* of the candidate phylum Cloacimonetes (WWE1) increased in all reactors and was the dominating bacterium at day 59. In particular, this bacterium showed a very high abundance in R1, which distinguished this reactor significantly from the other reactors in terms of microbial composition. Methane production and the reactor slurry characteristics were monitored in the digestion period. Generally all four reactors operated stably and showed rather similar characteristics. The average methane production in the reactors varied between 0.278 and 0.296 L gVS^-1^, with the lowest production in R1.

**Conclusions:**

This study showed that four parallel reactors co-digesting manure and fish waste silage operated stably during a startup phase. Several important Archaea and Bacteria degrading the protein-rich substrate were identified. In particular, microorganisms involved in syntrophic methane production seemed to be important. The detailed characterization of the microbial communities presented in this work may be useful for the operation of biogas plants degrading substrates with high concentrations of proteins.

## Background

Anaerobic digestion of organic materials from agriculture and industry may reduce local pollution and provide energy in the form of methane. Large amounts of organic materials are produced and disposed as waste every year. In Norway organic materials such as cattle manure and dead fish from fish farms are in large supply. In 2012 a loss of 27.4 million dead salmon was reported from Norwegian fish farms [1], and the total annual amount of organic waste in Norway is 1.45 million tons [2].

During anaerobic digestion organic materials are converted to methane and carbon dioxide plus small amounts of other gases by a microbial community through four main reactions: hydrolysis, acidogenesis, acetogenesis, and methanogenesis. The anaerobic degradation process is initiated by hydrolysis, where complex molecules like carbohydrates, lipids, and proteins are depolymerized into soluble compounds by a range of enzymes produced by the Bacteria. The hydrolyzed compounds are further fermented into acetate, propionate, butyrate, lactate, ethanol, methanol, ammonia, hydrogen, and carbon dioxide. Acetogenesis is the reaction in which acetate is produced from hydrogen and carbon sources by acetogenic Bacteria [3].

Methanogens belong to the Archaeal phylum Euryarchaeota [4], and methane is produced in the last step of the anaerobic process. The methane-producing microorganisms that usually dominate in biogas reactors are the acetoclastic methanogens [5]. The acetoclastic pathway is carried out by the order Methanosarcinales [6,7]. The primary substrate for methane production by the hydrogenotrophic methanogens is CO_2_ and H_2_, and this group consists of several methanogenic orders: Methanobacteriales, Methanococcales, and Methanomicrobiales [6,7]. An alternative methane production pathway, called syntrophic acetate oxidation, is known to take place in reactors with a high content of ammonia and fatty acids. The reaction includes conversion of acetate to H_2_ and CO_2_ by syntrophic acetate-oxidizing Bacteria, such as *Clostridium ultunense*, *Tepidanaerobacter acetatoxydans*, and *Syntrophaceticus schinkii*, followed by methane production by a hydrogenotrophic methanogen (for example, members of the orders Methanomicrobiales and Methanobacteriales) [8–11].

The acetogenic Bacteria and the methanogenic Archaea differ largely in terms of nutritional needs and sensitivity to environmental conditions [12]. Additionally, the methanogens have a slower growth rate than the acidogenic Bacteria [13], which in turn may result in accumulation of intermediate degradation products. A common reason for biogas reactor instability is failure to maintain the balance between these two groups of microorganisms [14].

The various complex anaerobic reactions that lead to methane formation are to a large extent performed through syntrophy between Bacteria and methanogenic Archaea. These syntrophic relationships provide the methanogens with their substrates and remove metabolic products from the acid-forming Bacteria [15]. Analyses of microbial communities have shown that elevated concentrations of ammonia in biogas reactors trigger the syntrophic acetate oxidation pathway, where acetate is transformed to CO_2_ and H_2_ before methane is produced by hydrogenotrophic methanogens [8,9]. The syntrophic degradation of other short chain fatty acids during anaerobic digestion has also been described [16,17], and several Bacterial strains and groups of methanogens are identified as having key roles in various syntrophic reactions.

Ensilaged fish waste contains large amounts of fat and protein [1], making it an energy-rich substrate that is suitable for biogas production. However, high inputs of fat and protein to a biogas reactor may cause accumulation of ammonia and fatty acids, potentially yielding unstable methane production and biogas reactor failure [15,18]. Generally, methanogens, and thus methane production, are inhibited by ammonia (NH_3_) formed in the process of protein degradation [19–21]. Long chain fatty acids (LCFAs) [22] and volatile fatty acids (VFAs) [23] formed from lipid degradation may also lead to inhibition.

The low pH of the ensilaged fish waste and the high concentrations of fat and protein make the substrate suitable for co-digestion with an alkaline organic material like cow manure. Co-digestion may improve the anaerobic digestion process by creating a better nutrient balance, diluting toxic compounds, and stimulating synergistic effects of microorganisms [24–26], and may possibly also increase the stability of the system and the methane production.

The startup is a critical phase in biogas reactors [13,27,28], and inoculum stability is highly important. Anaerobic microbial communities can adapt to high concentrations of ammonia and fatty acids [29], if a strategy of gradual acclimatization and proper adjustment of operational parameters such as substrate composition, organic loading rate (OLR), and hydraulic retention time (HRT) is applied [30]. During startup of a biogas reactor, many different groups of microorganisms with varying requirements for biochemical and physical conditions are introduced, and the initial one to three weeks are considered to be a reactor’s startup period [13]. Several experiments have dealt with startup dynamics in anaerobic digestion [13,31,32], but to our best knowledge, no metagenome analyses of microbial community structure in parallel continuously stirred tank reactors (CSTR) have been carried out.

The objectives of this study were to use metagenomic sequencing analysis to examine the microbial composition of a methane-producing inoculum, and to investigate the development of the inoculum through a stabilization period of 59 days in four parallel biogas reactors added protein-rich substrate under mesophilic conditions. The goal was also to compare the four reactors to investigate if the development of the microbial communities was similar in reactors running under the same conditions.

## Results and discussion

### Methane production and reactor slurry characteristics

**Figure 1:**
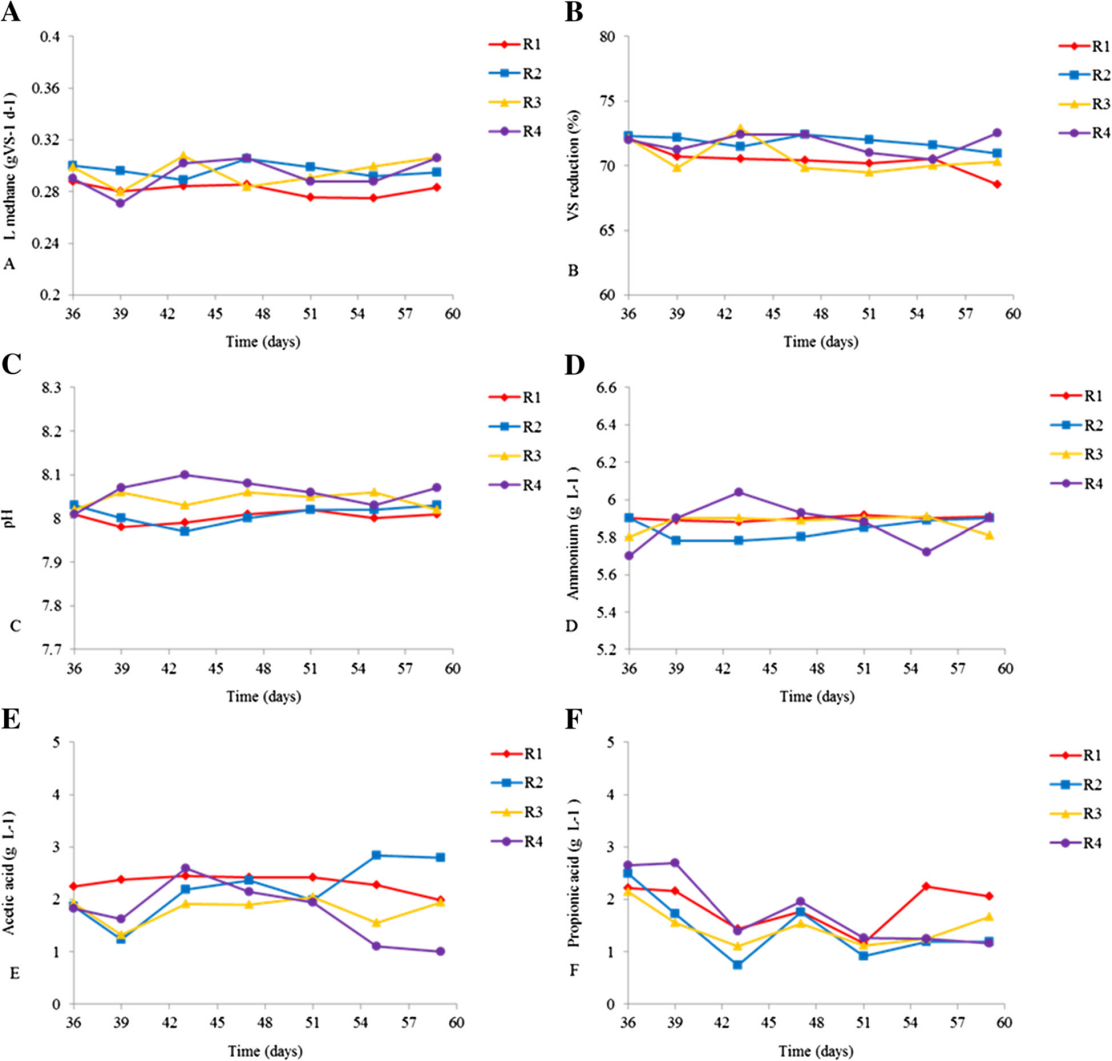
**Anaerobic process performance in R1, R2, R3, and R4 during 28 days of continuous operation (day 36 to day 59). A)** Methane productions, **B)** % volatile solid (VS) removal, **C)** pH values, **D)** NH_4_^+^ concentrations, **E)** acetic acid concentration, and **F)** propionic acid concentration.

The performance of four parallel biogas reactors during semicontinuous addition of fat and protein-rich materials (Table 1) was studied (Figure 1). The biogas volume and the CH_4_ and CO_2_ concentrations were measured once a day. In Figure 1A the average methane production is shown every fourth day. Although the CH_4_ production was quite similar in the four reactors, a somewhat lower CH_4_ production was observed from day 47 for R1. The average values of methane production in R1 and R2 were 0.282 (±0.039) and 0.297 (±0.042) L gVS^-1^, respectively (Figure 1A). These CH_4_ yields are in accordance with previous experiments on anaerobic co-digestion of the same substrates, where the yield was between 0.250 and 0.300 L CH_4_ gVS^-1^ [1]. The production of CO_2_ in the four reactors was on average between 0.142 and 0.161 L CO_2_ gVS^-1^ during the experiment (data not shown).

**Table 1:** **Chemical characterization of reactors’ substrate**

**Dry matter (%)**	Volatile solids* (%)	**Total nitrogen (g L** ^**-1**^ **)**	**Total carbon (g L** ^**-1**^ **)**	**Carbon/nitrogen**	**Acetic acid (g L** ^**-1**^ **)**	**Propionic acid (g L** ^**-1**^ **)**	**Ammonium (g L** ^**-1**^ **)**	**pH**
9.2	84.1	4.06	47.09	11.80	2.88	1.69	2.90	6.20

*Percentage of dry matter.

The NH_4_^+^, VFA, pH, and volatile solids (VS) reductions were measured every fourth day during the experimental period. The pH was around 8 in all the reactors during the entire period (Figure 1C). The average NH_4_^+^ concentrations were highest in R1, with a concentration of 5.90 (±0.08) g L^-1^, and lowest in R2, with a value of 5.84 (±0.042) g L^-1^ (Figure 1D), with corresponding NH_3_ concentrations in the range 0.67 to 0.75 g L^-1^ (data not shown). Previous experiments show that inhibition of methane production has been reported to take place at NH_3_ concentrations of 0.7 to 2.0 g L^-1^ [19,29,33,34]. On average, the concentrations of acetic acid in the reactors were lowest in R4 and highest in R1, ranging between 1.75 (±0.430) and 2.31 (±0.120) g L^-1^ (Figure 1E). The average concentrations of propionic acid varied between 1.43 (±0.482) and 1.86 (±0.351) g L^-1^ (Figure 1F), with the highest levels in R1 and the lowest in R2.

High levels of acetate are common in stable biogas reactors, while propionic acid has been reported to inhibit methanogenic activity in the range 0.8 g L ^-1^ [35] to 6 g L^–1^ [36]. Previous studies investigating methanogenic populations’ adaptation capabilities to NH_4_^+^, NH_3_, and VFAs have shown that methane production can be maintained in environments with high concentrations of these compounds [29]. The concentrations of NH_4_^+^ and VFAs observed in this study (Figure 1) were not alarmingly high, and the stable performance of the reactors suggest that the microbial communities in the reactors adapted to these conditions.

The amount of VS reduction (Figure 1B) supports the results of the other parameters measured, showing that the anaerobic degradation was somewhat lower in R1 than in the other reactors. The VS reduction in R1 decreased from 72.1 to 68.5 % from the startup of the continuous process to day 59, and the average VS reduction value in this reactor was 70.4 (±0.7)% (Figure 1B). In R2, R3, and R4, the VS removal values were quite similar and stable, with average values of 71.8 (±0.4), 70.6 (±1.0), and 71.7 (±0.7)%, respectively.

### Sequencing, coverage, and taxonomic richness

The results from pyrosequencing of the inoculum and the four reactors (day 59) before and after quality filtering are shown in Table 2. Unless otherwise specified, all percentages in the following text refer to the total number of reads in each of the filtered datasets.

**Table 2:** **Characteristics of metagenomic reads before and after quality filtering derived from DNA extracted from the four biogas reactors and their inoculum**

**Metagenome**	**Raw dataset**	**Filtered dataset**			
	**Number of reads**	**Number of reads**	**Reads (%)**	**Mean sequence length (bp)**	**Mean GC ratio (%)**
**R1**	245499	177017	72.10	413	43.08
**R2**	548434	390641	71.23	417	42.86
**R3**	182122	130610	71.72	410	43.75
**R4**	286008	205035	71.72	413	43.65
**IN**	241804	172150	71.19	409	43.10

**Figure 2:**
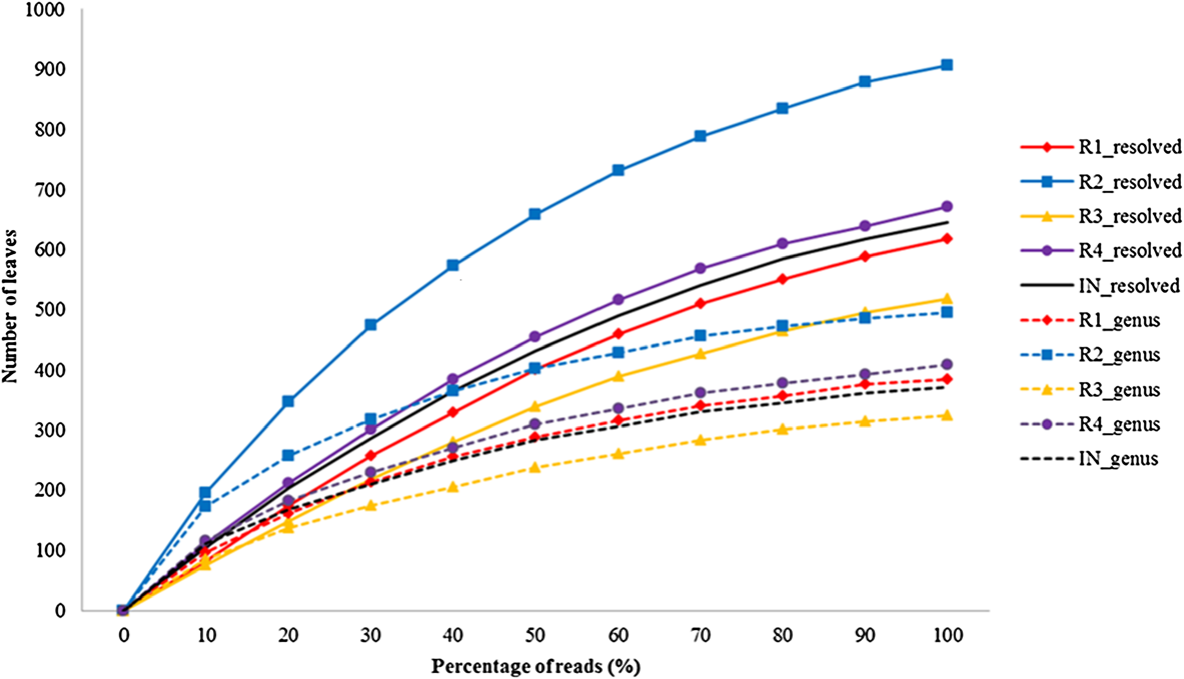
**Rarefaction curves of taxonomic richness in the samples at the genus and the fully resolved level in MEGAN.**

Rarefaction analysis in the program MEGAN was used to characterize the richness of taxa in the five metagenomes at the genus level and at the fully resolved level, where all species and strains were included (Figure 2). At the genus level, the curves were leveling off, indicating acceptable sampling and coverage of the richness in the samples. We detected from 324 (R3) to 496 (R2) genera (given as number of leaves in Figure 2). At the fully resolved level the number of taxa was in the range of 519 (R3) to 906 (R2). The richness in the samples was approximately proportional to the number of reads in the datasets (Table 2), and this may explain some of the variation in the number of taxa detected in the different samples. The high taxonomic richness shows that the samples harbor complex prokaryotic communities. The taxonomic richness in the inoculum (IN) was in the range of the reactor samples (R1 to R4).

Effective genome size (EGS) is a computational method to predict the average genome size, including multiple plasmid copies, inserted sequences, and associated phages and viruses, from short sequencing reads of metagenomes. EGS has been suggested as a link between the genome size and the functional repertoire of the metagenome; the greater the functional complexity, the greater the EGS [37]. In addition to the EGS values (Table 3), we calculated the probability (P) for detection of hits to a theoretical gene (X) of 1,000 bp. The expected number of hits to this gene X was calculated, assuming one copy number of this gene was present in all organisms in our communities. In the reactor samples (R1 to R4) the average EGS was 2.5. The slightly greater EGS in the inoculum (IN) than in the reactor samples may therefore indicate greater functional complexity in the inoculum compared to the reactor samples, which have experienced selective pressure in the 59-day stabilization period.

**Table 3:** **Effective genome size of the metagenomes**

**Metagenome**	**Effective genome size (Mbp)**	**Probability (P) of hitting gene X**	**Expected hits to gene X**
**R1**	2.2	0.000452462	80.0934268
**R2**	2.5	0.000404997	71.69129853
**R3**	2.6	0.000377457	66.81634238
**R4**	2.5	0.000394418	69.8187181
**IN**	3.2	0.000314795	55.72407323

### Taxonomic structure

**Figure 3:**
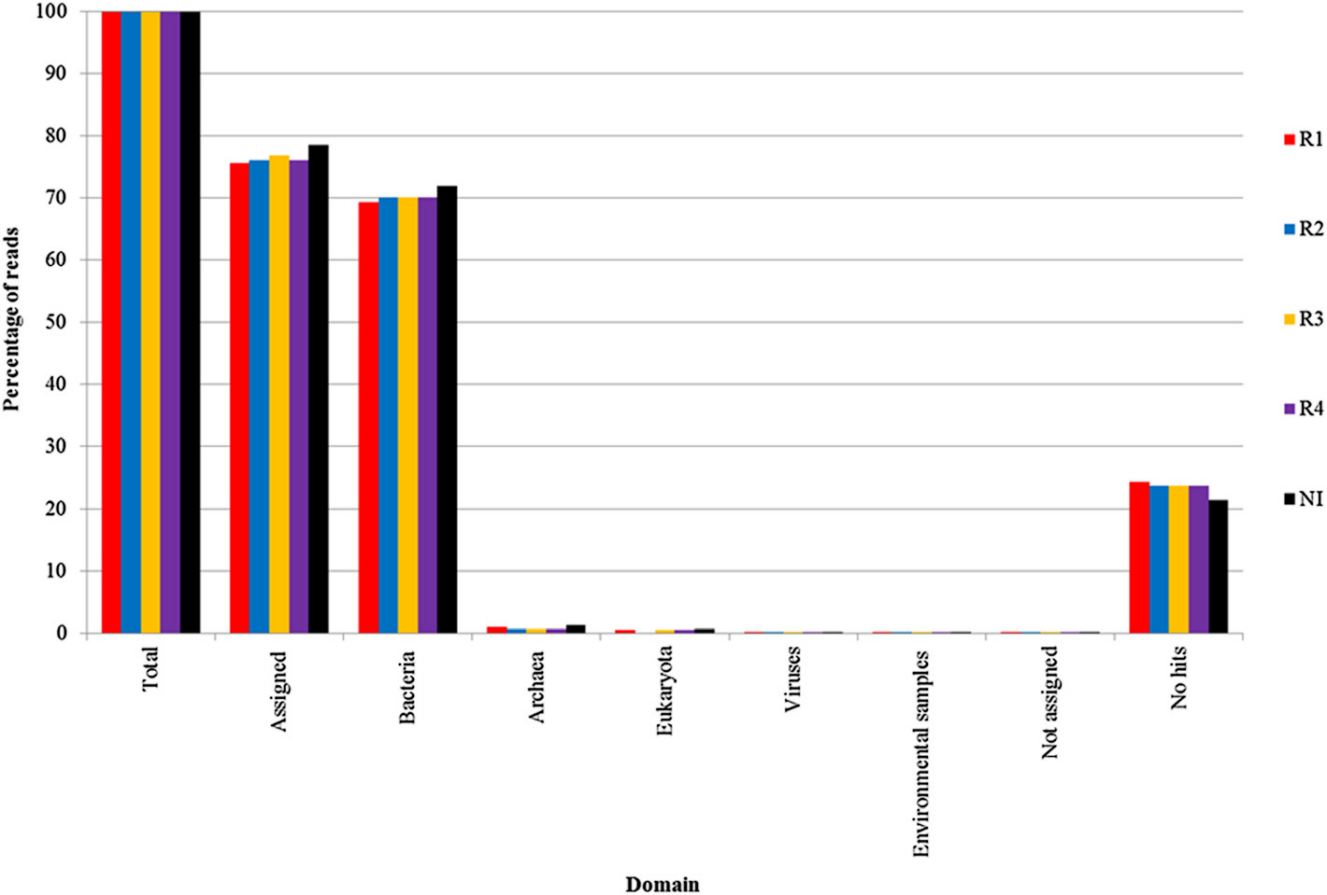
**Taxonomic distribution at the domain level in MEGAN.** Reads assigned at the domain level given as percentage of total reads in each metagenome. “No hits” are reads without hits in the BLAST search. “Not assigned” are reads with a hit in BLAST, but with no assignment to a taxon due to the settings in MEGAN. “Environmental samples” are reads with hits in other metagenome sequences with unknown biological classification.

The taxonomy at the domain level in the reactor samples and in the inoculum is shown in Figure 3. 75.64 to 78.48% of the total reads were assigned to taxa in MEGAN, while 21.46 to 24.31% were assigned to no hits. From 69.33% to 71.84% of the total reads were Bacterial, while 0.71% to 1.25% were assigned to Archaea. Although Archaea is usually less abundant than Bacteria in biogas reactors [38], the reads assigned to Archaea in our reactors are in the lower range of earlier reports. Typically, Archaea in biogas reactors is reported to be around 10% of the total reads [38–40]. However, other studies have reported Archaeal reads as low as 0.5% [41].

Eukaryota and viruses were also present in the metagenomes, representing from 0.44% to 0.58% and from 0.11% to 0.14%, respectively. Sample IN differed from the reactor samples by slightly greater percentages of reads assigned to Bacteria, Archaea, Eukaryota, and viruses, resulting in a corresponding reduction in reads with no hits.

**Figure 4:**
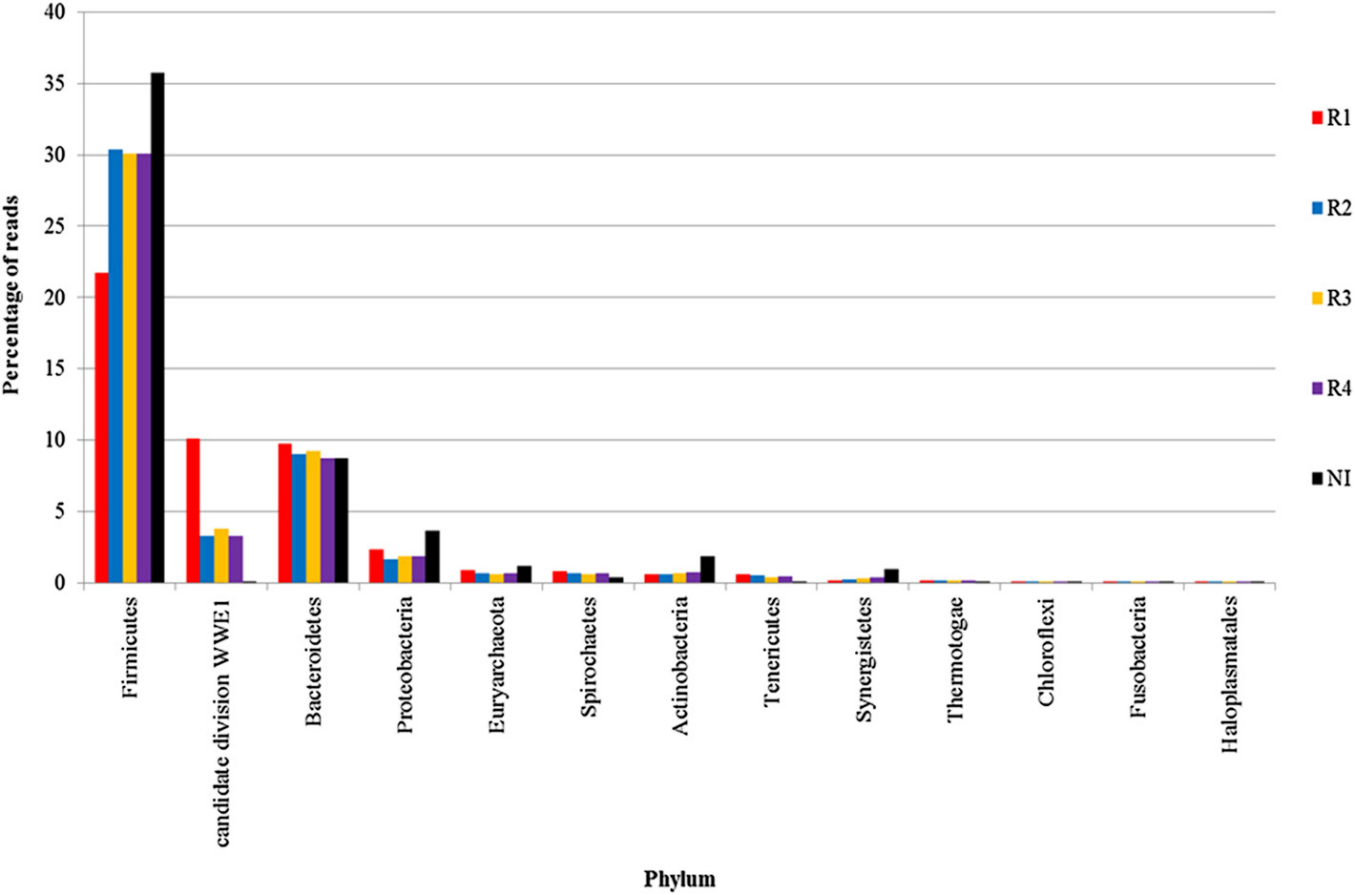
**Percentage of reads assigned to prokaryotic phyla with more than 0.1% of total reads assigned.**

A comparison of the taxonomic structures in the samples of phyla with more than 0.1% of the total number of reads assigned, in at least one metagenome, are given in Figure 4. The most abundant phyla in all the reactor samples were Firmicutes followed by Bacteroidetes and Cloacimonetes (WWE1). Together these phyla represented about 40 to 50% of all reads. This is in agreement with other investigations, which report that in nearly all microbial populations in methane-producing reactors, species from Firmicutes and Bacteroidetes are dominant [40,42]. It is therefore likely that these phyla are ubiquitous in all biogas reactors.

The structure in the inoculum (IN) differed from that of the reactor samples (R1 to R4) in several ways. IN harbored more of Firmicutes, Proteobacteria, Euryarchaeota, Actinobacteria, and Synergistetes, compared to the reactor samples. Comparison of the reactor samples only showed that R1 differed from the other samples. In particular, the abundance of Firmicutes is lower and the level of the *in silico* phylum, Cloacimonetes (WWE1), is greater in R1 than in the other reactor metagenomes. Proteobacteria, Euryarchaeota, Spirochaetes, and Tenericutes were also more abundant in R1 compared to the other reactors.

**Figure 5:**
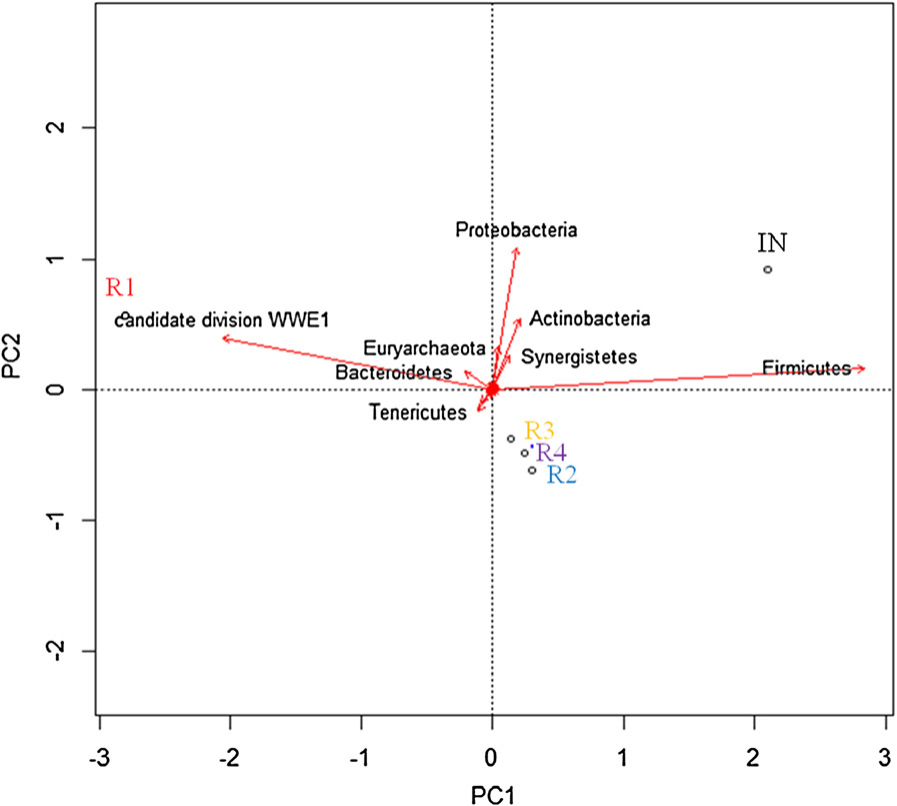
**PCA of phyla with Euclidean distance greater than 0.1 from origo.** Reads with no hits in the blast search and reads not assigned by MEGAN are excluded. The metagenomic parameters are represented by red arrows. Labels are shown for parameters with Euclidean distance over 0.1 from origo. All metagenome data were given as percentage of total reads.

Due to the complexity of the metagenomes, a principal component analysis (PCA) plot, at the phylum level, was constructed to view the clustering of the five samples (Figure 5). The reactor samples R2, R3, and R4 were highly similar and clustered closely in the lower right quadrant, while sample R1, located in the upper left quadrant, differed in several ways from the other reactor metagenomes. As expected, the inoculum sample separated from all the reactor samples in the PCA plot and was positioned in the upper right quadrant. The abundances of Firmicutes and Cloacimonetes (WWE1) were the most important parameters for positioning of the samples along the first principal component (PC1). Firmicutes, Actinobacteria, and Synergistetes all had positive scores along PC1, indicating that the samples placed on the right section of the PCA plot (IN, R2,R3,R4) had relatively high abundances of these taxa compared to sample R1. Proteobacteria and Euryarchaeota have positive scores along PC1 but also strong positive scores at PC2, indicating a greater abundance of these phyla in R1 and IN compared to the other samples. The separation of R1 from the other reactor samples (R2, R3, and R4) is mainly due to its high content of Cloacimonetes (WWE1) but also of Bacteroidetes.

**Figure 6:**
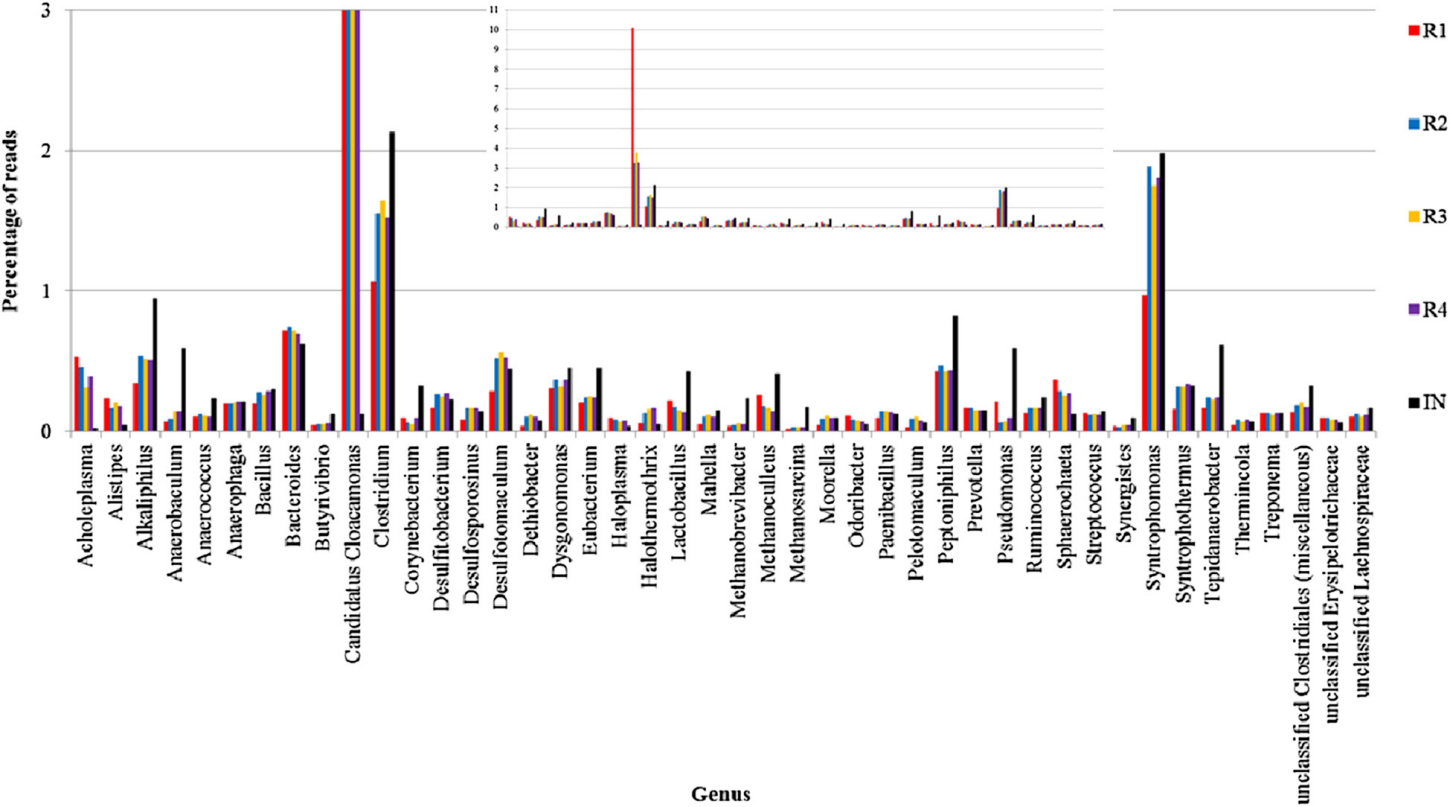
**Percentage of reads assigned to the 44 genera with more than 0.1% reads assigned.** Insert shows full scale of the Y-axes.

Of the 324 to 496 genera detected in the rarefaction analysis (Figure 2), 44 genera were characterized as highly abundant as each of them harbored ≥0.1% of the reads in one or more of the metagenomes (Figure 6). *Candidatus Cloacimonas* (of the phylum Cloacimonetes (WWE1)) [43] is the most abundant genus in the reactor samples, where it represented from 3.26% (R2) to 10.10 % (R1) of the reads. The abundance of this taxon in the inoculum (IN) is considerably lower (0.12%). An increasing abundance of phylum Cloacimonetes (WWE1) over longer anaerobic digestion periods has been observed previously [44]. The species *Candidatus Cloacimonas acidaminovorans* has not been cultivated, and the complete genome was reconstructed from a metagenomic analysis of a biogas reactor digesting municipal wastewater [45]. *In silico* proteome analysis indicated that this bacterium derived most of its carbon and energy from the fermentation of amino acids. The gene content suggests *Candidatus Cloacimonas acidaminovorans* to be a syntroph producing H_2_ and CO_2_ from formate, and this strain is probably present in many anaerobic digesters [45].

Altogether 25 genera of the phylum Firmicutes were among the highly abundant genera (Figure 6). The two genera, *Clostridium* and *Syntrophomonas*, each accounted for about 1 to 2% of the reads in all the five metagenomes. The 23 remaining highly abundant Firmicutes genera were: *Alkaliphilus*, *Anaerococcus*, *Bacillus*, *Dethiobacter*, *Butyrivibrio*, *Desulfitobacterium*, *Desulfosporosinus*, *Desulfotomaculum*, *Eubacterium*, *Halothermothrix*, *Lactobacillus*, *Mahella*, *Moorella*, *Paenibacillus*, *Pelotomaculum*, *Peptoniphilus*, *Ruminococcus*, *Streptococcus*, *Syntrophothermus*, *Tepidanaerobacter*, unclassified Clostridiales (miscellaneous), unclassified Erysipelotrichaceae, and unclassified Lachnospiraceae. The following six genera of the phylum Bacteroidetes were also among the highly abundant taxa (Figure 6): *Alistipes*, *Bacteroides*, *Dysgonomonas*, *Odoribacter*, *Porphyromonas*, and *Prevotella*. Three genera of the phylum Synergistetes (*Anaerobaculum*, *Anaerophaga*, *Synergistes*), three genera of the Archaeal phylum Euryarchaeota (*Methanobrevibacter*, *Methanoculleus*, *Methanosarcina;* all methane producers) and two genera of the phylum Spirochaetes (*Treponema* and *Sphaerochaeta*) were also highly abundant in the metagenomes. In addition, we detected the following genera as highly abundant (phylum indicated in brackets): *Acholeplasma* (Tenericutes), *Corynebacterium* (Actinobacteria), *Haloplasma* (unclassified Bacteria), and *Pseudomonas* (Proteobacteria).

The most abundant genus of Firmicutes in the biogas reactors was *Clostridium*. In general, Clostridia are known to be involved in the hydrolytic digestion of macromolecular compounds in the first step of a fermentation process, and therefore play a crucial role in biogas production [38,46,47].

**Figure 7:**
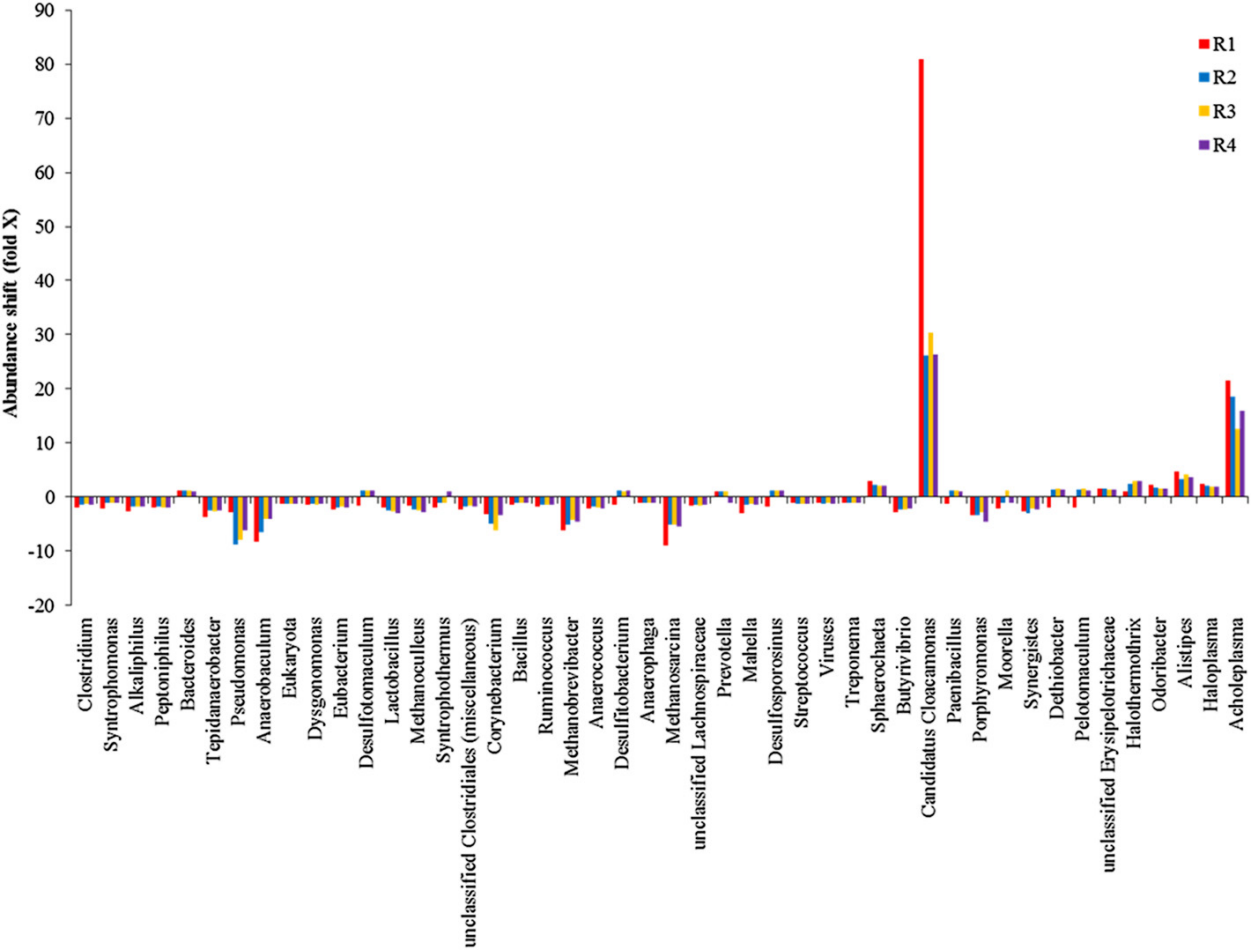
**Abundance shift compared to inoculum at the genus level.** This figure shows the fold change in abundant genera (>0.1% in one or more metagenomes) in the reactor samples compared to the inoculum. Fold change values less than 1 were replaced by the negative of their inverse.

The taxonomic analysis revealed great diversity of highly abundant genera in all samples. Still the high abundance of *Candidatus Cloacimonas*, *Clostridium*, and *Syntrophomonas* indicated a major role of these genera in the biogas reactors and in the inoculum. Abundance shifts in the reactor samples compared to the inoculum at the genus level are illustrated in Figure 7. The predominant change is the large increase of *Candidatus Cloacimonas* in the reactor samples, especially R1, indicating an important role of this genus in the reactors. There is also a relatively large increase in the abundance of *Acholeplasma*, while *Pseudomonas*, *Anaerobaculum*, *Corynebacterium*, *Methanobrevibacter*, and *Methanosarcina* are among the genera most reduced in their abundance compared to the inoculum.

**Figure 8:**
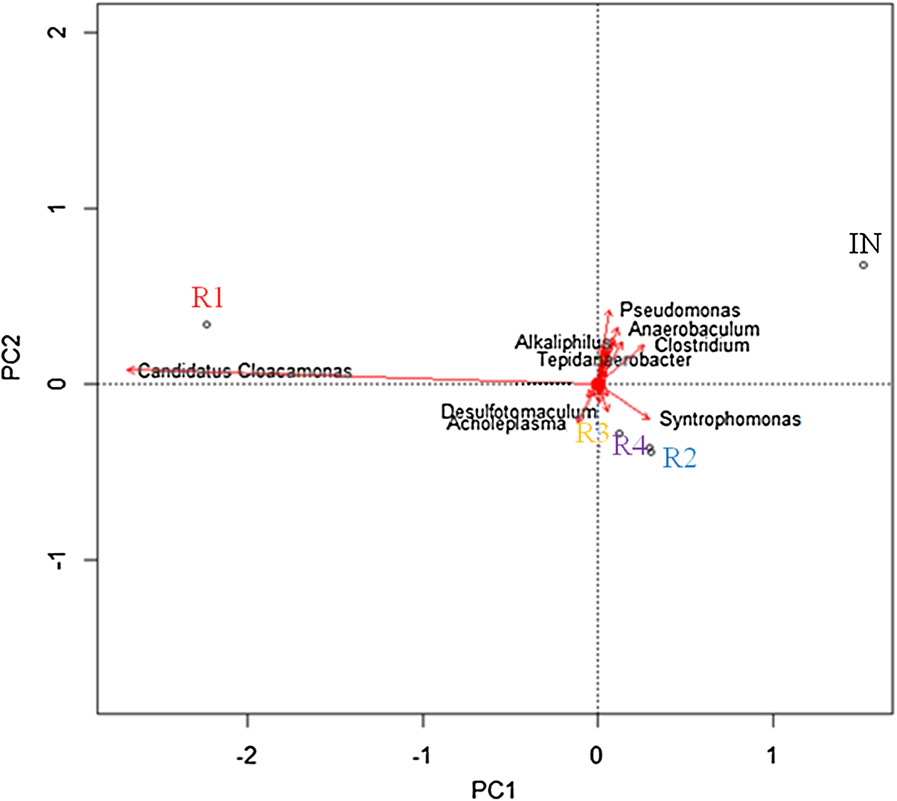
**PCA of genera with Euclidean distance from origo greater than 0.1.** Reads with no hits in the BLAST search and reads not assigned by MEGAN are excluded. The metagenomic parameters are represented by red arrows. Labels are shown for parameters with Euclidean distance over 0.1 from origo. All metagenome data were given as percentage of total reads.

To further study the clustering of the metagenomes, a PCA plot at the genus level was constructed. All genera were included, but reads with no hits were excluded (Figure 8). When the genus level was used, the metagenomes of R2, R3, and R4 clustered more closely than in the PCA plot at the phylum level (Figure 6). The overall clustering pattern of the samples at the genus level is however similar to the clustering detected at the phylum level. This supports consistency in the clustering analysis using PCA and shows that the same clustering is expressed at two quite different taxonomic levels of these complex metagenomes. Figure 8 shows that it is the significantly higher abundance of the genus *Candidatus Cloacimonas* in R1 that gives the major contribution to R1's separation from samples R2, R3, and R4. Inspection of the MEGAN charts showed that all reads of this genus were further assigned to the strain *Candidatus Cloacimonas acidaminovorans* str. Evry. As suggested from a reconstruction of the complete genome [45], this uncultivated strain is probably a syntrophic bacterium that is present in many anaerobic digesters.

In a previous study carried out by Kovács *et al.* [48], changes in the composition of the microbial community were detected through the use of a highly parallel SOLiD® (Sequencing by Oligo Ligation and Detection) next generation DNA sequencing on samples from fed-batch reactors fed with a low C/N ratio substrate. It was found that the bacterium *Candidatus Cloacimonas acidaminovorans* disappeared when the reactors were added a protein monosubstrate with a C/N ratio of 3. This bacterium is not capable of producing polyamines and a number of other cofactors. In our experiments we observed an increase in the abundance of *Candidatus Cloacimonas acidaminovorans* in all reactors (Figure 6).

The Firmicutes genus *Syntrophomonas* strongly influenced the clustering of R2, R3, and R4 in the lower right quadrant. It should be noted that the abundance of this genus is much less in R1 compared to the other reactors and the inoculum. Inspection of the MEGAN charts revealed the strain *Syntrophomonas wolfei* as the predominant *Syntrophomonas* in all the five metagenomes. *S. wolfei* is a Gram-negative bacterium isolated from anaerobic environments like aquatic sediments or sewage sludge [49]. This organism is able to beta-oxidize saturated fatty acids (C4 to C8 fatty acids) anaerobically to acetate, or to acetate and propionate, in the presence of a syntrophic partner [50]. Fatty acid degradation also leads to production of H_2_, which is consumed by a syntrophic methanogenic partner (the Methanomicrobiales strain *Methanospirillum hungatei* has been reported) [51]. The syntrophic H_2_ transfer mechanism from *Syntrophomonas* to the methanogen is probably mediated by format because H_2_ cannot diffuse rapidly enough to account for the level of methane synthesis in methanogenic cultures [52]. Another synergist known to be involved in syntrophic acetate oxidation under high NH_4_^+^ concentrations, *Tepidanaerobacter acetatoxydans* [10,11], was detected in our biogas reactors, with higher abundance in R2, R3, and R4 than in R1. Potential methanogenic syntrophic partners to *Syntrophomonas* were also present in the metagenomes. The methanogenic genus *Methanospirillum* was present with low density in all the reactor samples in this study (data not shown), but the genera *Methanoculleus* and *Methanobrevibacter* (Figure 6) were abundant. Overall, the high abundance of syntrophic Bacteria indicates that syntrophic methane production is important in these reactors.

### Methanogenesis and subsystems of metabolism

The methanogenic Archaea play a major role in the global carbon cycle by carrying out the final methane-producing step in the anaerobic degradation of organic materials. Methanogens typically thrive in environments where all electron acceptors other than CO_2_ are depleted.

Inspection of the MEGAN charts of *Euryarchaeota* at the genus level revealed great diversity in all metagenomes. The genus *Methanoculleus* of the order Methanomicrobiales, followed by the genus *Methanobrevibacter* of the order Methanobacteriales (both orders are known to produce methane from H_2_ and CO_2_) were the most abundant in all the samples (Table 4). *Methanosarcina* and *Methanosaeta* of the order Methanosarcinales were present, but the abundance was significantly lower. Members of the genus *Methanoculleus* are among the most prevalent methanogens found in wastewater, sewage bioreactors, and landfills [53]. All reads of the genus *Methanoculleus* in the MEGAN analyses were further assigned to the species *Methanoculleus marisnigri* JR1. This organism has all genes required for methanogenesis from hydrogen and CO_2_ [54]_._ In addition this organism can use formate and secondary alcohols such as propanol and butanol as electron donors in methanogenesis. The high abundance of Methanomicrobiales in the reactor samples is in consistence with the relative high VFA levels in the reactors, which indicate high hydrogen production. The high levels of acetate in the reactors are in accordance with the abundance of the methanogenic genus *Methanosarcina* (*M. acetivorans, M. barkeri,* and *M. mazei*). These methanogens are known to be capable of using all the three degradation pathways for methane formation (acetate, methyl, and hydrogen). Acetate cleavage has been regarded to be dominated by Methanosarcinaceae at high acetate concentrations and by Methanosaetaceae at low acetate concentrations [55]. Absence of Methanosaetaceae is also correlated with acetate oxidation [55].

**Table 4:** **Percentage of reads assigned to the most abundant methanogenic genera**

**Metagenome**	***Methanoculleus***	***Methanobrevibacter***	***Methanosarcina***	***Methanosaeta***
**R1**	0.264	0.038	0.019	0.021
**R2**	0.183	0.046	0.034	0.017
**R3**	0.164	0.057	0.034	0.011
**R4**	0.145	0.052	0.031	0.018
**IN**	0.417	0.236	0.171	0.017

**Figure 9:**
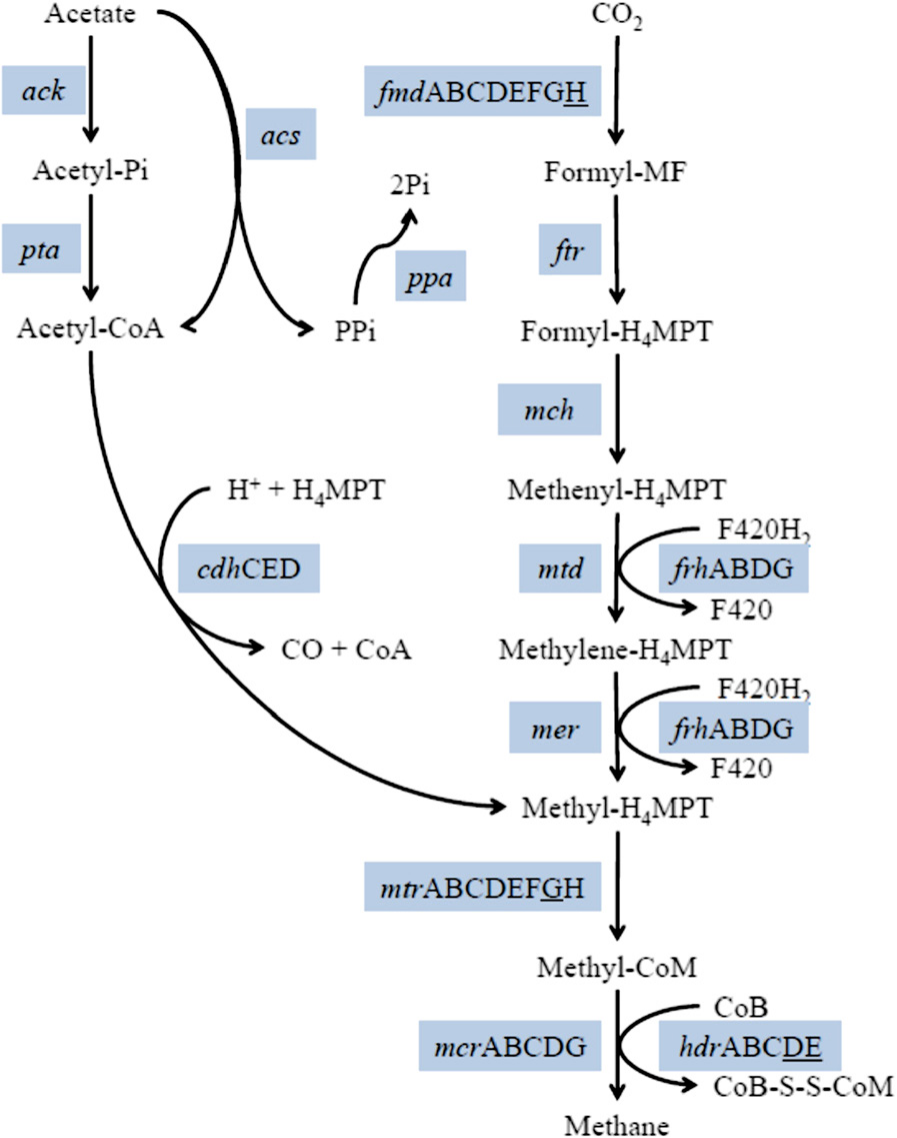
**The methanogenesis pathway.** Enzymes are shown in blue boxes. Subunits missing in all our datasets (R1, R2, R3, R4, and IN) after search against the KO database at MG-RASTare underlined. Abbreviations used in the figure are Acetyl-Pi: acetyl phosphate; ack: acetate kinase; acs: acetyl-CoA synthetase; cdh: acetyl-CoA decarbonylase/synthase; CO: carbon monoxide; CoA: coenzyme A; CoB: coenzyme B; CoB-S-S-CoM: coenzyme M 7-mercaptoheptanoylthreonine-phosphate heterodisulfide; F420: coenzyme F420; fmd: formylmethanofuran dehydrogenase; Formyl-H4MPT: 5-formyl-5,6,7,8-tetrahydromethanopterin; Formyl-MF: formylmethanofuran; frh: coenzyme F420 hydrogenase; ftr: formylmethanofuran-tetrahydromethanopterin N-formyltransferase; H4MPT: 5,6,7,8-tetrahydromethanopterin; hdr: heterodisulfide reductase; mch: methenyltetrahydromethanopterin cyclohydrolase; mcr: methyl-coenzyme M reductase; mer: 5,10-methylenetetrahydromethanopterin reductase; Methenyl-H4MPT: 5,10-methenyl-5,6,7,8-tetrahydromethanopterin; Methyl-CoM: methylcoenzyme M; Methylene-H4MPT: 5,10-methylenetetrahydromethanopterin; Methyl-H4MPT: 5-methyl-5,6,7,8-tetrahydromethanopterin; mtd: methylenetetrahydromethanopterin dehydrogenase; mtr: tetrahydromethanopterin S-methyltransferase; ppa: inorganic diphosphatase; pta: phosphate acetyltransferase.

The abundance of *Methanoculleus*, *Methanobrevibactor, Methanosarcina*, and *Methanosaeta* in the reactor samples indicates that the methane production was carried out by both hydrogenotrophic and acetoclastic methanogenesis. Figure 9 shows the results from a KEGG analysis of functional enzymes involved in methane production. Enzymes for methane formation from both CO_2_ and hydrogen, and acetate were present in the reactors. These results support the assumption that methane was formed from both hydrogenotrophic and acetoclastic reaction pathways in the reactors.

**Figure 10:**
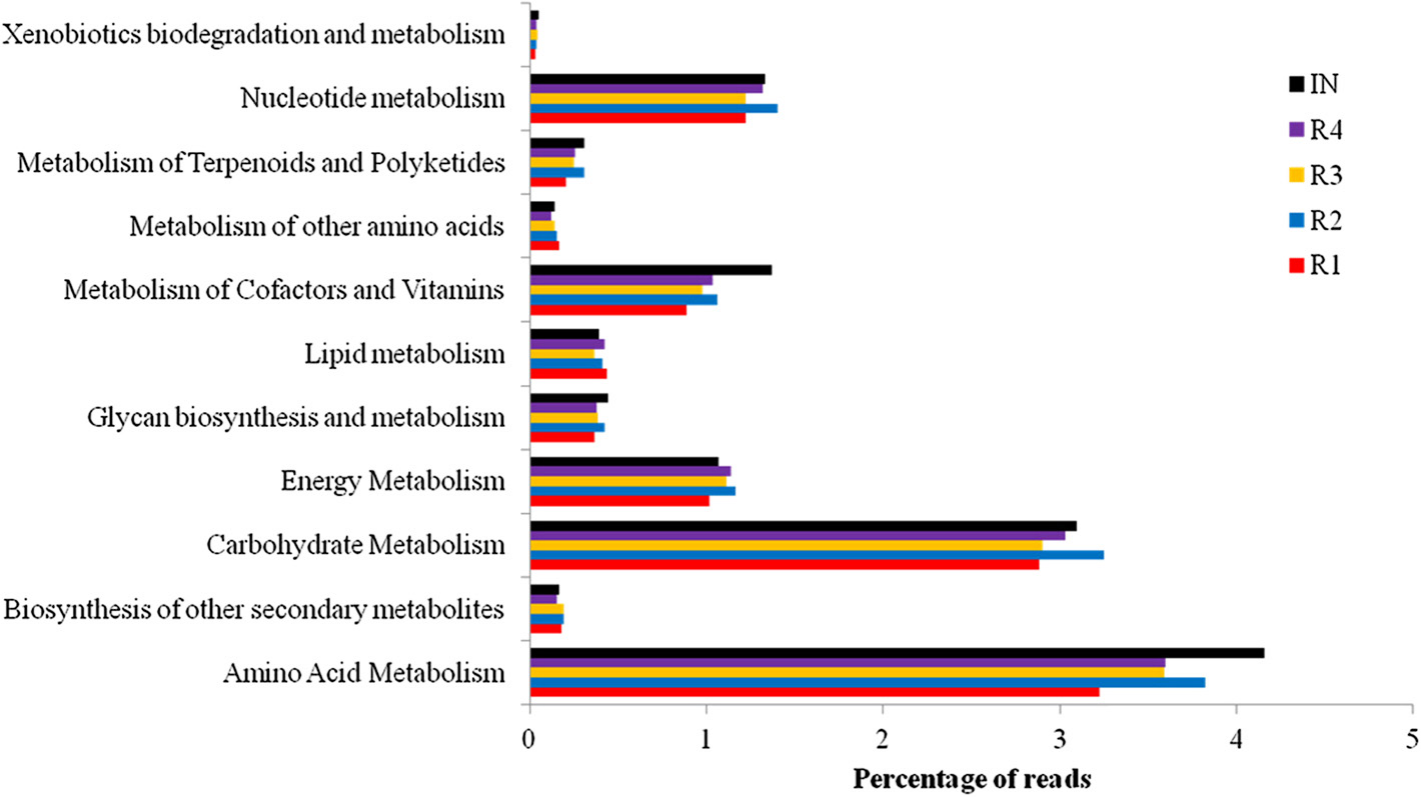
**Reads assigned to level 2 metabolism subsystems at MG-RAST (KO database).**

Figure 10 shows the results from the KEGG analysis of metabolic systems that are related to methane production, including metabolism of amino acids, energy, carbohydrates, nucleotides, lipids, cofactors, vitamins, polyketides, terpenoids, glycan, and xenobiotics. These metabolic activities are associated with the conversion of biomass into methane during anaerobic fermentation. The results show that a large amount of reads are distributed among amino acid metabolism and carbohydrate metabolism. This observation is consistent with the finding that many species found in the samples are involved in amino acid and carbohydrate digestion. The amount of protein in the fish waste silage that was added to our reactors during the experiment is 15% (ww), and the high content of protein in the substrate is consistent with the abundant reads for enzymes involved in the amino acid metabolism. In a previous study, Li *et al.* [41] used fat- and protein-rich food waste as a biogas substrate, and they found that a significant amount of reads were obtained for the processes involved in the protein degradation pathway. Among the genes involved in the carbohydrate metabolism, those that degrade cellulose are particularly important for the efficient breakdown of substrates such as co-manure. The high percentage of reads assigned to carbohydrate metabolism and the abundance of the Firmicutes phylum and *Clostridium* genus in our reactors demonstrate the importance of carbohydrate and cellulose degradation by the anaerobic microbial community.

The result on carbohydrate metabolism is in agreement with previous investigations [47,56,57], and the evaluation of metabolic pathways shows that the capabilities of metabolism varied somewhat in the different reactors. Enzymes related to metabolism of amino acids and cofactors and vitamins were highest in the IN sample. Among the reactor samples, R1 in general had the fewest reads assigned to metabolic subsystems, while the highest number of reads assigned to subsystems was detected in R2 and R4.

Methane production in R1 was somewhat lower than in the other reactors. The propionic acid concentration in this reactor was somewhat higher than in the other reactors. This, together with the high abundance of *Candidatus Cloacimonas acidaminovorans* in R1 may have had an impact on this reactor’s somewhat lower metabolic capabilities. The high prevalence of the bacterium might have been an inhibiting factor in R1.

## Conclusions

Taxonomic and functional studies of inoculum and reactor samples showed that the microbial consortium changed over time in the four reactors during the digestion phase. The results show that the microbial community in the four biogas reactors after 59 days of operation was different from the microbial composition in the inoculum. A greater functional complexity was detected in the inoculum compared to the reactor samples. Microbial communities tend to become more specific and less complex over time when degrading the same substrate. The results showed that the microbial composition developed quite similarly in three of the four parallel reactors during the experiment.

Bacteria from the phylum Firmicutes were most abundant in the reactors, followed by the phyla Bacteroidetes and Proteobacteria. In particular, the species from genera *Clostridium* and *Syntrophomonas* play key roles in the initial degradation of protein, cellulose, and other polysaccharides. These results were further supported by gene functional annotation, where we detected many enzymes involved in protein degradation and carbohydrate metabolism. The dominant methanogens present in the reactors were from Methanomicrobiales*,* and the most prevalent genus appeared to be *Methanoculleus. Methanobrevibacter, Methanosarcina*, and *Methanosaeta* were also detected in the samples. These methanogens use versatile substrates and contain both acetotrophic and hydrogenotrophic pathways for methane production.

The microbial composition in one reactor (R1) differed from those of the others, especially in relation to the high prevalence of the Bacterium *Candidatus Cloacimonas acidaminovorans*. This reactor also showed lower average methane production and VS removal than the other three reactors, and this might be linked to the difference in microbiology. One possible theory for the dissimilarity is that the high density of the *Candidatus Cloacimonas acidaminovorans* in R1 may have had a negative impact on the syntrophic relationships between Bacteria and methanogens in this reactor. R1 had, in addition to very high values of Cloacimonetes (WWE1), also a low density of Firmicutes*,* a phylum consisting of many important syntrophic members of the class Clostridia (e.g. *Syntrophomonas*). The reactor also had a low density of the syntrophic acetate-oxidizing bacteria *Tepidanaerobacter acetatoxydans*. It should be noted that the 454 sequencing in this study was not done in replicate, mainly due to the cost of the analysis. While the method is known to be very reproducible [58], future studies of parallel reactors should ideally also include replicate sequencing.

This study showed that four parallel reactors co-digesting manure and fish waste silage operated stably during a startup phase. Clear changes in the microbial population were seen in all four reactors, the most pronounced being the increased abundance of *Candidatus Cloacimonas acidaminovorans*. Additionally, several important Archaea and Bacteria degrading the protein-rich substrate were identified. In particular, microorganisms involved in syntrophic methane production seemed to be important. These results give leads for the design of well-functioning microbial communities for biogas plants digesting similar substrates.

## Materials and methods

### Inoculum, substrate, and reactors operation

Slurry from a biogas reactor co-digesting a mix of 19% (v/v) fish waste silage and 81% cow manure [1] was used as the inoculum (IN). In a previous study the high amount of amount of fish waste silage led to process inhibition due to overloading of protein and fat. The slurry from this reactor was kept without any addition of substrate for 50 days, until startup of the present experiment. The chemical composition of the inoculum was measured in triplicate samples, and had the following characteristics: pH =8.1 (±0.09), NH_4_^+^ (g L^-1^) =5.5 (±0.08), DM (%) =6.3 (±0.06), VS of DM (%) =73.6 (±0.51). The substrate used was a mix of 87% cow manure and 13 % fish waste silage (v/v). The substrate chemical composition was measured in triplicate samples, and its characteristics are shown in Table 1.

To four 10-L continuously stirred tank reactors, designated R1, R2, R3, and R4, were added 2.55 L inoculum (day 0). From day one substrate was added to the reactors every day until the effective reactor volume was 8 L. The amount of substrate added each day was calculated from the reactors’ increasing effective volume and 30 days HRT (for example, based on a reactor with 2.55 L effective volume and an HRT of 30 days: 2.55 L/30 d =85 mL substrate/reactor/day). At day 36 the reactors were fed with 266.7 mL substrate, yielding a final effective volume of 8 L. From day 36 to day 59 (28 days), the reactors were fed at a fixed rate of 266.7 mL substrate/reactor/day (this was based on reactors with 8 L effective volume, and an HRT of 30 days: 8 L/30 d =266.7 mL substrate/reactor/day). The same amount of reactor slurry was removed (prior to substrate addition) each day to maintain the volume at 8 L. The reactors were operated anaerobically at 37°C with a stirring speed of 150 rpm. The total carbon and nitrogen in the substrate was determined in single samples, and the carbon:nitrogen (C/N) ratio was calculated. Approximately 20 g inoculum was collected at day 0, and 50 g slurry from each of the four reactors (R1, R2, R3, and R4) were collected at day 59, and stored frozen (-20°C) in 50 mL Nunc centrifuge tubes prior to DNA extraction.

### Chemical analysis procedures

The content of DM and VS, and the pH in the inoculum, the reactor slurries, and in the substrate were determined according to standardized methods [59–61] every fourth day, in triplicate samples.

The NH_4_^+^ and VFA concentrations in the inoculum and in the reactor slurries were determined every fourth day. The concentration of NH_4_^+^ was determined in triplicate samples by use of an ammonium selective electrode (Thermo Scientific Orion ISE/NH4). Samples for NH_4_^+^ analysis were diluted (1:10) in distilled water and measured at 20°C and supplemented with an ionic strength adjustor (28.7 g glacial acetic acid L ^-1^ and 53.6 g magnesium acetate L^-1^), using 10 mL of ionic strength adjustor per 100 mL of sample, for stabilization of NH_4_^+^. The NH_3_ concentrations were calculated based on the NH_4_^+^ concentrations. The average deviations between the triplicate samples (not shown) were <0.5. Samples for VFA (acetic acid and propionic acid) analysis were centrifuged (13,000 rpm) and filtrated (0.45 μm) prior to analysis. The concentrations of VFAs were determined in single samples, by use of a Rezex RFQ Fast Acid H + (8%) 100 × 7.8 mm HPLC (Phenomenex, Torrance, CA, USA), operated at a temperature of 85°C, with an Ultimate 3000RS column and UV detection at 210 nm (Dionex, Sunnyvale, CA, USA ). The samples were diluted with sulfuric acid (8 μL total) before analysis.

The elemental composition of carbon, hydrogen, and nitrogen was determined in the reactors’ substrate by combustion using a LECO CHN-1000 instrument (St. Joseph, MI, USA).

The biogas was collected in 25-L Tedlar bags (Tedlar® Gas Sampling Bag, Sigma-Aldrich, St. Louis, MO, USA). CH_4_ and CO_2_, as a percentage of the gas volume of samples, were measured once a day with a GA2000 Landfill Gas Analyzer (Geotechnical Instruments Ltd., UK). The total gas production rate volume (L/d) was calculated from flow measurements (rate 300 cm^3^/min) as follows: (pump-number/60 seconds * 300 cm^3^-min) / (1000 mL).

### DNA extraction from reactor samples

All samples for DNA extraction were collected at the same time and treated in exactly the same way. In order to achieve homogeneous and representative samples, the inoculum and the reactor slurries were thoroughly stirred before and during sampling. The samples were collected in 100-mL plastic bottles and frozen. The frozen samples of the inoculum (IN) and reactors (R1 to R4) were slowly thawed before the total genomic DNA was extracted from duplicate subsamples using a FastDNA SPIN Kit for soil (MP Biomedicals, Santa Ana, CA), according to the producer’s instruction. Lysis and homogenization of the samples were performed in a Bertin Technologies (Rockville, MD) Precellys 24 system, for 2 × 20 seconds at speed 5400. Each subsample was eluted from the columns with 100 μL DNase/pyrogen-free water (DES). The combined eluates were purified using a Wizard® DNA Clean-Up System (Promega, Madison, WI) and finally eluted from the Wizard column with 50 μl DES. The DNA purity and concentrations were measured in a NanoVue spectrophotometer and Qubit assay using the Qubit® 2.0 Fluorometer. The DNA quality and chain length were inspected in 1.2% agarose (Biozyme RESult, LE General Purpose Agarose) gel in 1× Tris-acetate-EDTA with added 20 μL SYBR Safe DNA gel stain, 10,000 concentration in DMSO (Life Technologies, Grand Island, NY) to a final gel volume of 200 mL. DNA extracts were added using TrackIt™ Cyan/Yellow Loading buffer (Invitrogen) to a final volume of 10 μL. Three microliters of Trackit™ 100 bp DNA Ladder (Invitrogen) were used. The agarose gel was run at 100 V for 90 minutes. Images of the gel were made using a KODAK Gel Logic 212 Imaging System for inspection of the chain length prior to 454 pyrosequencing.

### 454 pyrosequencing

Sample preparation and sequencing of extracted DNA were performed at the High Throughput Sequencing Centre at CEES, University of Oslo [62], according to standard 454 GS FLX Titanium protocol. The five samples were tagged, mixed, and sequenced on a 70 × 75 format PicoTiterPlate™ on a GS FLX Titanium instrument. The sequence data have been submitted to the NCBI database (http://www.ncbi.nlm.nih.gov) under BioProject accession number PRJNA261310.

### Quality filtering

The complete datasets were analyzed with PRINSEQ [63] to determine the sequence quality scores. For each sample we performed quality filtering to remove low quality reads (reads containing ≥10 ambiguous bp, homopolymers of ≥10 bp, and sequence length <100 bp) in mothur v.1.25.1 [64]. The trimmed files were checked for artificial replicates using cdhit-454 with standard settings [65]. The cleaned files were analyzed with PRINSEQ before the files were uploaded at the Bioportal computer service [66] for Blast X against the NCBI non-redundant Protein database (ncbiP-nr). The maximum expectation value was set to 10^-3^, and a maximum of 25 alignments were reported per hit.

### Effective genome size

The effective genome size (EGS) for each metagenome was estimated according to the method developed by Raes *et al.* [37], using the constants a =18.26, b =3650, and c =0.733. A protein reference database containing the 35 single copy COGs in question was downloaded from STRING (v. 9.0) [67]. BlastX was conducted at the freely available Bioportal computer service [68]. The sampling probability of a random universal single copy gene (1000 bases) and expected number of reads detected were calculated according to Beszteri *et al.* [69].

### Taxonomic classification

The BlastX output files were analyzed according to NCBI taxonomy in the program MEGAN, version 4 [70,71] with default LCA parameters (Min Score: 35, Top Percent: 10.0, and Min Support: 5). All taxa were enabled.

### Principal component analysis

The PCA plots were created using the vegan library in R [72] as previously described [73]. The ordination was based on reads assigned to the phylum and to the genus level in MEGAN. All metagenome data were given as a percentage of total reads.

### Metabolic annotation

The metagenomic reads were assigned to subsystems on the MG-RAST server [74] (version 3.3.9) [75]. The KEGG Orthology (KO) reference database was used. The maximum expectation value was set to 10^-5^, the minimum alignment length was set to 50 bases, and the minimum percentage identity was set to 50%. We used the same settings to search the metagenomes for key genes involved in methanogenesis.

## Abbreviations

Acetyl-Pi: acetyl phosphate
ack: acetate kinase
acs: acetyl-CoA synthetase
cdh: acetyl-CoA decarbonylase/synthase
CO: carbon monoxide
CoA: coenzyme A
CoB: coenzyme B
CoB-S-S-CoM: coenzyme M 7-mercaptoheptanoylthreonine-phosphate heterodisulfide
DES: DNase/pyrogen-free water
DM: dry matter
DMSO: dimethyl sulfoxide
F420: coenzyme F420
fmd: formylmethanofuran dehydrogenase
Formyl-H4MPT: 5-formyl-5,6,7,8-tetrahydromethanopterin
Formyl-MF: formylmethanofuran
frh: coenzyme F420 hydrogenase
ftr: formylmethanofuran-tetrahydromethanopterin N-formyltransferase
H4MPT: 5,6,7,8-tetrahydromethanopterin
hdr: heterodisulfide reductase
mch: methenyltetrahydromethanopterin cyclohydrolase
mcr: methyl-coenzyme M reductase
mer: 5,10-methylenetetrahydromethanopterin reductase
Methenyl-H4MPT: 5,10-methenyl-5,6,7,8-tetrahydromethanopterin
Methyl-CoM: methylcoenzyme M
Methylene-H4MPT: 5,10-methylenetetrahydromethanopterin
Methyl-H4MPT: 5-methyl-5,6,7,8-tetrahydromethanopterin
mtd: methylenetetrahydromethanopterin dehydrogenase
mtr: tetrahydromethanopterin S-methyltransferase
ppa: inorganic diphosphatase
pta: phosphate acetyltransferase
VFA: volatile fatty acid
VS: volatile solids
